# Familial pediatric Peutz–Jeghers syndrome with recurrent intussusception: case report and literature review

**DOI:** 10.3389/fped.2026.1768468

**Published:** 2026-03-11

**Authors:** Sondes Sahli, Bochra Aziza, Nada Sghairoun, Asma Slimani, Nadia Boujelbene, Rim Missaoui, Zohra Rahal, Said Jlidi

**Affiliations:** 1 Department of Pediatric Surgery, Children Hospital Tunis, Tunis, Tunisia; 2 Universite de Tunis El Manar Faculte de Medecine de Tunis, Tunis, Tunisia; 3 Institut Salah Azaiez, Tunis, Tunisia; 4 Universite de Tunis El Manar Faculte des Sciences de Tunis, Tunis, Tunisia; 5 Departement of Pediatric Surgery of Beja, Beja, Tunisia

**Keywords:** children, intestinal intussusception, intestinal polyposis, Peutz–Jeghers syndrome, surgery

## Abstract

**Background:**

Peutz–Jeghers syndrome (PJS) is a rare autosomal dominant disorder characterized by mucocutaneous pigmentation and gastrointestinal hamartomatous polyposis, predisposing affected individuals to recurrent small bowel intussusception and increased cancer risk.

**Case presentation:**

We report two siblings with genetically confirmed PJS who presented with intestinal obstruction due to small bowel intussusception. The first, a 13-year-old girl, had necrotic ileal intussusception caused by a large polyp requiring segmental resection. Her younger brother had jejuno-jejunal intussusception secondary to multiple intraluminal polyps, managed by manual reduction and polyp extraction. Histopathological examination revealed characteristic hamartomatous Peutz–Jeghers polyps with villous architecture and arborizing bundles of compact smooth muscle. Genetic analysis confirmed a pathogenic STK11/LKB1 mutation in both patients. At 5-year follow-up, both remained asymptomatic under regular endoscopic and imaging surveillance.

**Conclusion:**

PJS should be suspected in children presenting with recurrent small bowel intussusception, particularly with mucocutaneous pigmentation or a positive family history. Early diagnosis, bowel-preserving surgery, and long-term multidisciplinary follow-up are key to preventing recurrence and malignant transformation.

## Introduction

Peutz–Jeghers syndrome (PJS) is a rare autosomal dominant disorder characterized by mucocutaneous lentiginosis and gastrointestinal hamartomatous polyposis. The condition predisposes affected individuals to gastrointestinal (particularly small intestinal, but also gastric and colonic) as well as extraintestinal (especially pancreatic, breast, and potentially pulmonary) malignancies (1, 2). Recurrent intestinal obstruction, particularly due to small bowel intussusception in younger patients, represents a serious complication (3). We report a new familial case and highlight the clinical, histopathological, and therapeutic features of this syndrome, whose pathogenesis remains incompletely understood.

## Case presentation

### Case 1

**Figure 1:**
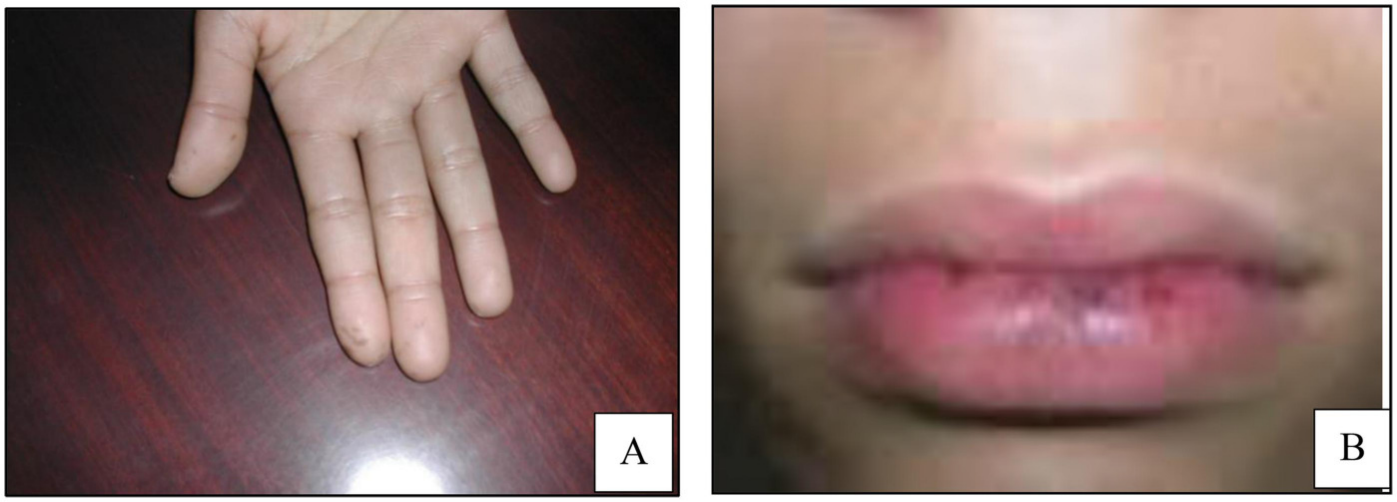
Melanin pigmentation of the fingertips **(A)** and thelips **(B)**.

A 13-year-old girl, whose father had a history of colonic polyposis, was admitted to the pediatric surgery department with signs of acute intestinal obstruction, including abdominal pain, distension, and vomiting. Physical examination revealed multiple lentiginous macules on the lips and fingertips (Figures 1A,B), characteristic of Peutz–Jeghers syndrome.

**Figure 2:**
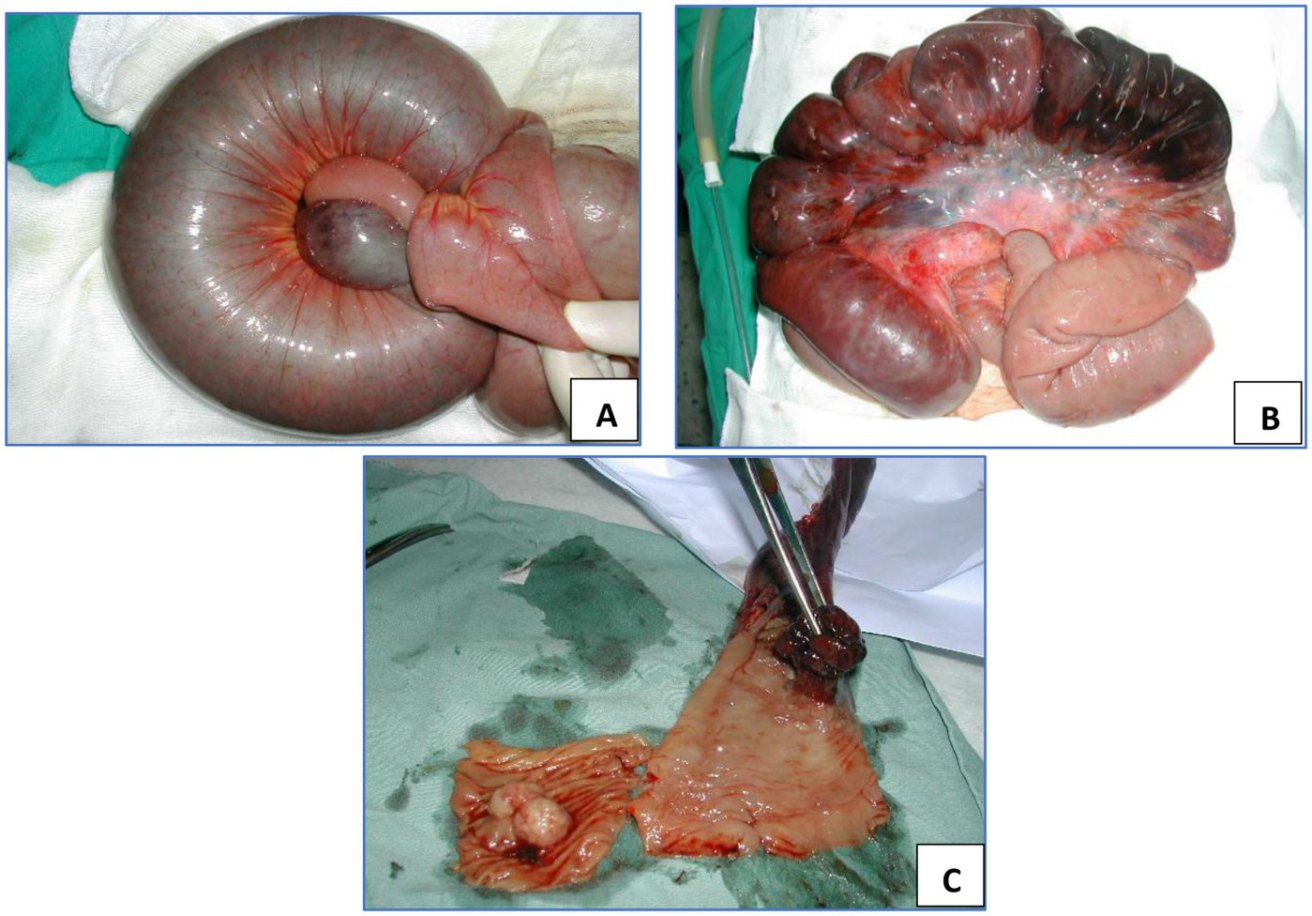
**(A)** Jejuno-jejunal intussusception with a congested and edematous small-bowel loop. **(B,C)** After opening of the intussusception, a necrotic pedunculated jejunal polyp measuring 4 cm in length is identified as the lead point.

Abdominal ultrasonography demonstrated significant small bowel distension with a target-like mass suggestive of intussusception. Exploratory laparotomy revealed a 40-cm segment of necrotic ileal intussusception containing a pedunculated intraluminal polyp measuring approximately 4 cm in length (Figures 2A–C). Segmental small bowel resection with immediate end-to-end anastomosis was performed. The postoperative course was uneventful, and the patient was discharged on postoperative day 7.

**Figure 3:**
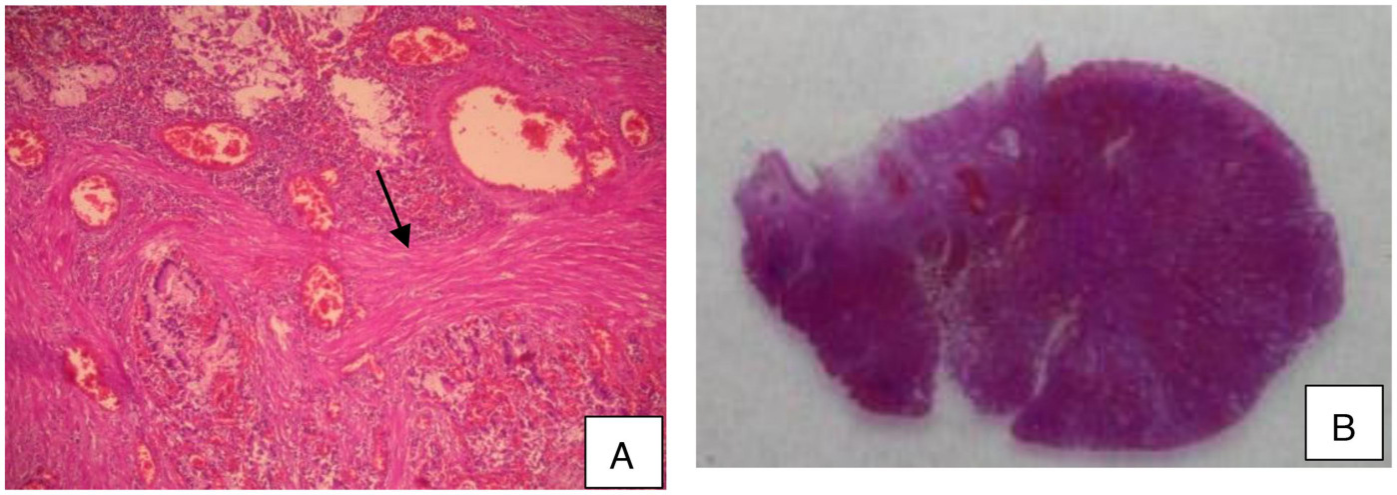
Peutz–Jeghers polyp. **(A)** Papillary villous architecture with an arborizing smooth muscle core (arrow). **(B)** Lobular arrangement of epithelial components separated by smooth muscle bundles (H&E stain; **A** × 40, **B** × 100).

Histopathological examination of the resected specimen revealed a hamartomatous polyp with a papillary villous architecture and an arborizing smooth muscle core lined by normal mucosal epithelium, confirming the diagnosis of a Peutz–Jeghers polyp (Figures 3A,B).

Subsequent gastrointestinal evaluation included upper gastrointestinal endoscopy, which demonstrated small polyps located in the gastric cardia and fundus that were left *in situ* due to their typical hamartomatous appearance without atypical or malignant features, and a 6-mm sessile rectal polyp located 10 cm from the anal verge, which was removed endoscopically. Small bowel follow-through was normal. Histological examination of the rectal polyp confirmed a hamartomatous Peutz–Jeghers polyp without dysplasia.

At 5-year follow-up, the patient remained asymptomatic, with no evidence of recurrent intussusception or new polyp formation on serial imaging and endoscopic surveillance.

### Case 2

The 13-year-old younger brother of the previously described patient was admitted with recurrent episodes of paroxysmal abdominal pain accompanied by bilious vomiting. On physical examination, multiple lentiginous macules were noted over the nasal bridge and palms. Abdominal palpation revealed localized tenderness and a palpable paraumbilical mass.

**Figure 4:**
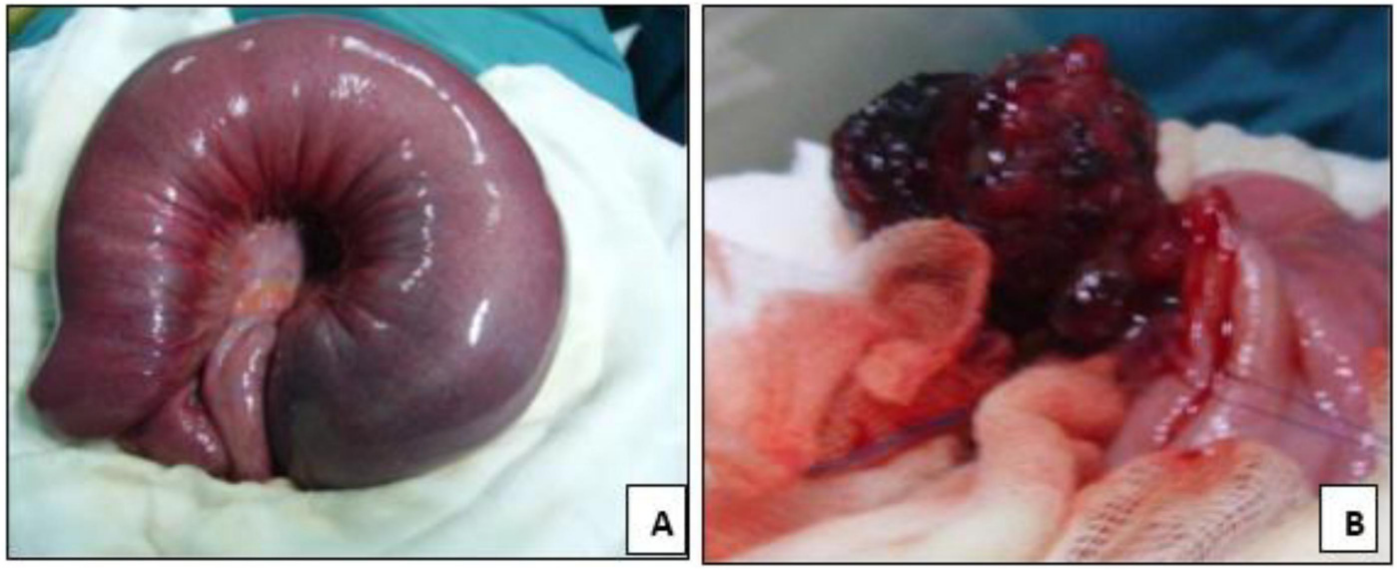
**(A)** Jejuno-jejunal intussusception showing a congested and edematous intestinal loop before reduction. **(B)** After manual reduction, an intraluminal jejunal polyp is identified as the lead point of the intussusception.

Abdominal ultrasonography demonstrated a concentric “target” sign consistent with small bowel intussusception. Exploratory laparotomy revealed a 160-cm segment of jejunal intussusception containing multiple intraluminal polyps (Figure 4A). Gentle reduction of the intussuscepted bowel was performed, followed by enterotomy and complete extraction of the polyps (Figure 4B). The affected intestinal segment was viable and did not require resection. The postoperative recovery was uneventful, and the patient was discharged on the seventh postoperative day in good condition.

**Figure 5:**
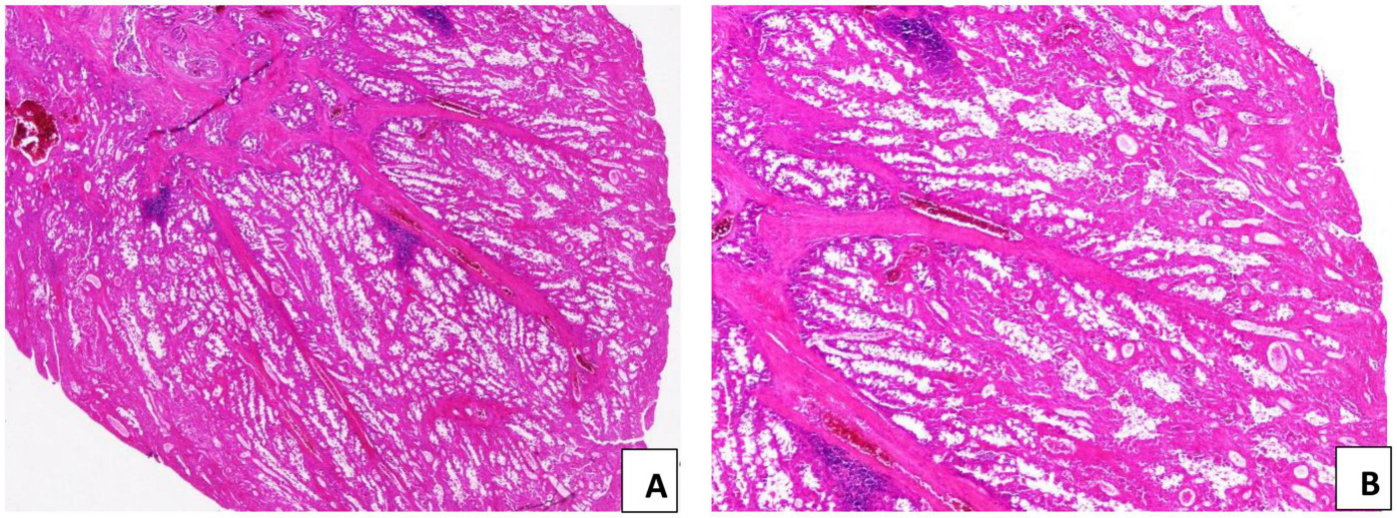
Peutz-Jeghers polyp. **(A)** Papillary villous architecture with an arborizing smooth muscle core **(B)** The epithelial component is arranged in a lobular configuration, separated by smooth muscle cores (H&E, **A** × 40, **B** × 100).

Histological examination shows the same features as those previously described and confirms the diagnosis of a Peutz–Jeghers polyps (Figure 5). Subsequent upper gastrointestinal endoscopy and small bowel follow-through were normal, with no additional lesions detected.

**Figure 6:**
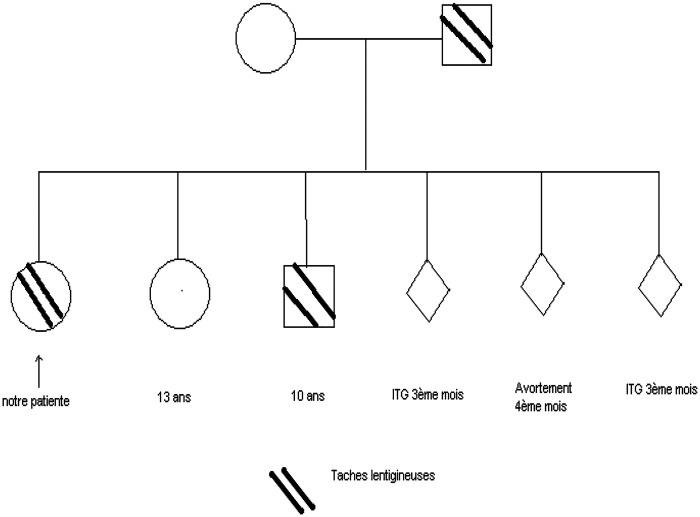
The family pedigree of the patient with Peutz–Jeghers syndrome illustrates affected and unaffected members.

Genetic testing in both siblings confirmed the presence of a constitutional pathogenic variant in the STK11/LKB1 tumor suppressor gene, establishing the molecular diagnosis of Peutz–Jeghers syndrome (Figure 6). Table 1 illustrates the chronological timeline of clinical events of the two siblings with Peutz–Jeghers syndrome.

**Table 1:** Chronological timeline of clinical events in two siblings with Peutz–Jeghers syndrome.

Age/time point	Patient 1 (older sister)	Patient 2 (younger brother)
Family history	Father with history of colonic polyposis	Same family history
Age at presentation	13 years	9 years
Presenting symptoms	Acute abdominal pain, vomiting, abdominal distension consistent with intestinal obstruction	Recurrent paroxysmal abdominal pain with bilious vomiting
Physical examination	Mucocutaneous lentiginous macules on the lips and fingertips	Lentiginous macules on the nasal bridge and palms; palpable paraumbilical mass
Initial imaging	Abdominal ultrasonography showed small bowel distension with a target-like mass suggestive of intussusception	Abdominal ultrasonography demonstrated a concentric “target” sign consistent with small bowel intussusception
Intraoperative findings	Necrotic ileal intussusception involving a 40-cm bowel segment with a single pedunculated intraluminal polyp measuring approximately 4 cm	Jejuno-jejunal intussusception involving a 160-cm segment with multiple intraluminal polyps
Surgical management	Segmental small bowel resection with primary end-to-end anastomosis	Manual reduction of intussusception followed by enterotomy and complete extraction of polyps; bowel preserved
Postoperative course	Uneventful recovery; discharged on postoperative day 7	
Histopathology	Hamartomatous Peutz–Jeghers polyp with papillary villous architecture and arborizing smooth muscle core	
Additional GI evaluation	Upper endoscopy: small gastric polyps left *in situ*; 6-mm rectal polyp removed; small bowel follow-through normal	Upper gastrointestinal endoscopy and small bowel follow-through normal
Genetic testing	constitutional pathogenic variant STK11/LKB1 identified	Same constitutional pathogenic variant STK11/LKB1 identified
Follow-up outcome	Asymptomatic at 5-year follow-up with no recurrence	

## Discussion

Peutz–Jeghers syndrome is inherited in an autosomal dominant fashion, with non-sporadic cases typically demonstrating a history in a first-degree relative, as in our patients whose father was affected. It is characterized by the presence of pigmented macules on the lips, oral mucosa, and digits, along with multiple hamartomatous polyps throughout the gastrointestinal tract (4). The condition is caused by pathogenic variants in the STK11/LKB1 tumor suppressor gene located on chromosome 19p13.3, which plays a crucial role in cell growth regulation and tumor suppression (5, 6). Familial aggregation is common, as illustrated in our cases, where the father had a history of colonic polyposis.

Clinical manifestations usually arise during childhood or adolescence, with most patients diagnosed before the age of 20. Abdominal pain due to intermittent intussusception is the most frequent symptom, while gastrointestinal bleeding may result in chronic anemia. Less commonly, patients present with vomiting, acute bowel obstruction, or intestinal necrosis (7, 8). Mucocutaneous lentigines are typically asymptomatic but serve as an important diagnostic marker, appearing early in life and remaining stable over time without malignant potential (9).

Gastrointestinal polyps in PJS are most frequently located in the small intestine, particularly the jejunum and ileum, with occasional gastric or colonic involvement. Rare cases have been reported in the biliary or urinary tracts (10). Histologically, these polyps are hamartomatous, with a branching smooth muscle core covered by normal epithelium. A minority may harbor adenomatous changes, which contribute to the elevated cancer risk observed in these patients. Malignant transformation can occur in both gastrointestinal and extraintestinal sites, highlighting the importance of long-term surveillance (11, 12).

Recurrent intussusception is a major complication of PJS, often necessitating surgical intervention (13). Conservative strategies, such as endoscopic polypectomy (including the use of double-balloon enteroscopy and intraoperative enteroscopy) or limited enterotomy for polyp removal, are preferred to preserve bowel length and prevent short bowel syndrome (14, 15, 17–20). Extensive resections are reserved for nonviable segments or inaccessible lesions (16, 18). Lifelong surveillance with periodic endoscopic and imaging assessments is essential to identify new polyps, prevent complications, and detect malignant transformation (8). Beyond gastrointestinal involvement, Peutz–Jeghers syndrome is associated with an increased risk of extraintestinal malignancies, most notably pancreatic and breast cancers. Owing to their low incidence in childhood, current guidelines, including those of the European Hereditary Tumour Group (18), do not recommend routine pancreatic cancer screening. Conversely, affected individuals, particularly women, should be enrolled in high-risk breast cancer surveillance programs in adulthood, typically starting at approximately 30 years of age with annual breast MRI and/or mammography.

Recent advances in diagnostic modalities have significantly improved the detection and management of Peutz–Jeghers syndrome, particularly in pediatric patients. Video capsule endoscopy and magnetic resonance enterography are increasingly used for non-invasive evaluation of small bowel polyps, allowing earlier detection and risk stratification while minimizing radiation exposure. Therapeutically, device-assisted enteroscopy techniques, including double-balloon and single-balloon enteroscopy, have enabled effective endoscopic polypectomy of deep small bowel lesions, reducing the need for repeated surgical interventions. Laparoscopic-assisted and intraoperative enteroscopic polypectomy further support bowel-preserving strategies in patients with extensive polyposis or recurrent intussusception (19, 20).

Current recommendations emphasize lifelong surveillance beginning in childhood, with periodic upper and lower gastrointestinal endoscopy and small bowel evaluation to prevent complications and detect malignancies at an early stage. Multidisciplinary follow-up involving pediatric gastroenterologists, surgeons, radiologists, and genetic counselors is essential, particularly in familial cases, to optimize outcomes and guide screening of at-risk relatives.

The favorable outcomes observed in our patients, with no recurrence of symptoms over a 5-year follow-up period, highlight the importance of early diagnosis, appropriate surgical decision-making, and structured long-term surveillance in pediatric Peutz–Jeghers syndrome.

Table 2 summarizes the clinical features, the management strategy and outcomes of Pediatric Peutz–Jeghers Syndrome Cases in the recent Literature.

**Table 2:** Summary of pediatric Peutz–Jeghers syndrome cases in the recent literature.

Study	Year	Number of patients	Age (years)	Main clinical features	Management strategy	Follow-up (years)	Outcomes
Achatz et al. (3)	2017	Review including ∼40 pediatric cases	<18 years	Early-onset polyposis, abdominal pain, intussusception	Surveillance and early polyp removal	4 years	Early screening reduces complications.
Kirakosyan et al. (14)	2020	18 Children	5–17 years	Recurrent small-bowel obstruction, intussusception	Double-balloon enteroscopy, endoscopic polypectomy	3.2 years	Low recurrence rate.
Xu et al. (9)	2023	Pediatric subset within 566 cases (≈120)	6–17 years	Abdominal pain, bleeding, recurrent intussusception	Endoscopic and surgical resection	4.6 years	Good long-term outcomes
Verma et al. (16)	2024	8 patients	4–16 years	Recurrent small-bowel intussusception	Limited resection or laparoscopic reduction	3 years	No recurrence
Dofuku et al. (17)	2025	25 children	5–17 years	Multiple jejunal polyps, recurrent obstruction	Endoscopic ischemic polypectomy	2.8 years	100% Technical success 0.3% Complication
Goneidy et al. (18)	2025	14 patients	7–15 years	Recurrent intussusception from large polyps	Laparoscopic-assisted polypectomy	3.5 years	Excellent outcomes
Zhongguo Dang et al. (15)	2024	Review of 37 pediatric cases	4–17 years	Small-bowel obstruction, melena	Endoscopic + surgical hybrid	4 years	No recurrence
Our cases		Two siblings, both	13 years	Recurrent jejuno-ileal intussusception, mucocutaneous pigmentation, confirmed STK11 mutation	Case 1: segmental resection (necrosis)	5 years	No recurrence
					Case 2: enterotomy + polyp extraction		

## Conclusion

Peutz–Jeghers syndrome is an uncommon hereditary disorder that should be considered in pediatric patients presenting with small bowel intussusception and characteristic mucocutaneous pigmentation. Early diagnosis, supported by genetic testing, enables appropriate family screening and surveillance. Bowel-preserving surgical and endoscopic techniques are preferred to minimize morbidity, and lifelong multidisciplinary follow-up is essential to detect recurrent polyps and reduce the risk of malignant transformation.

## Patient perspective

The siblings' families initially experienced anxiety due to sudden abdominal emergencies and uncertainty about the cause. They were relieved after timely surgery, bowel preservation, and confirmation of Peutz–Jeghers syndrome, with long-term follow-up planned. The parents appreciated clear communication, involvement in decision-making, and multidisciplinary care, which minimized stress and fostered trust in the healthcare team.

## Data Availability

The original contributions presented in the study are included in the article/Supplementary Material, further inquiries can be directed to the corresponding author.

## References

[1] Giardiello FM Brensinger JD Tersmette AC Goodman SN Petersen GM Booker SV Very high risk of cancer in familial Peutz-Jeghers syndrome. Gastroenterology. (2000) 119(6):1447–53. 10.1053/gast.2000.20228 11113065

[2] Hearle N Schumacher V Menko FH Olschwang S Boardman LA Gille JJP Frequency and spectrum of cancers in the Peutz-Jeghers syndrome. Clin Cancer Res. (2006) 12(10):3209–15. 10.1158/1078-0432.CCR-06-0083 16707622

[3] Achatz MI Porter CC Brugières L Druker H Frebourg T Foulkes WD Cancer screening recommendations and clinical management of inherited gastrointestinal cancer syndromes in childhood. Clin Cancer Res. (2017) 23:107–14. 10.1158/1078-0432.CCR-17-0790 28674119

[4] Sun Q Wang X-Y Guo G-J Meng L-M Ning S-B. Global research landscape of Peutz-Jeghers syndrome and successful endoscopic management of intestinal intussusception in patients with recurrent laparotomies. World J Gastrointest Surg. (2024) 16(8):2702–18. 10.4240/wjgs.v16.i8.2702 39220083 PMC11362939

[5] Daniell J Plazzer JP Perera A Macrae F. An exploration of genotype-phenotype link between Peutz-Jeghers syndrome and STK11: a review. Fam Cancer. (2017) 17:421–7. 10.1007/s10689-017-0037-3 28900777

[6] Kazubskaya TP Kozlova V Filippova M Тrofimov EI Belev N Sokolova I. Hereditary syndromes associated with polyps and the development of malignant tumors in children. Oncopediatrics. (2015) 2:384–95. 10.15690/onco.v2.i4.1465 27070770

[7] Miyachi T Tanaka N Endo K Fujishima F Sasaki H Nagao M A case of Peutz-Jeghers syndrome with repeated small intestinal intussusception successfully treated by intraoperative endoscopic polypectomy. Nihon Shokakibyo Gakkai Zasshi. (2013) 110(6):1014–21. 23739734

[8] Aytin YE Türkyılmaz Z. A rare cause of mechanical intestinal obstruction due to small bowel intussusception: “A solitary Peutz-Jeghers type hamartomatous polyp”. Ulus Travma Acil Cerrahi Derg. (2022) 28(6):879–83. 10.14744/tjtes.2021.34560 35652870 PMC10443007

[9] Xu Z-X Jiang L-X Chen Y-R Zhang Y-H Zhang Z Dong Z-W Clinical features, diagnosis, and treatment of Peutz-Jeghers syndrome: experience with 566 Chinese cases. World J Gastroenterol. (2023) 29(10):1627–37. 10.3748/wjg.v29.i10.1627 36970589 PMC10037245

[10] Lokhmatov MM Budkina TN Oldakovsky VI Tupylenko AV Ibragimov SI. Syndrome: diagnostic and therapeutic possibilities of modern intraluminal endoscopy on the example of our own clinical observation. Pediatr Pharmacol. (2016) 13:395–8. 10.15690/pf.v13i4.1614

[11] Tacheci I Kopacova M Bures J. Peutz-Jeghers syndrome. Curr Opin Gastroenterol. (2021) 37(3):245–54. 10.1097/MOG.0000000000000718 33591027

[12] Xu Z Gu G. Cancer risk of Peutz-Jeghers syndrome and treatment experience: a Chinese medical center. Clin Colon Rectal Surg. (2023) 36(6):406–14. 10.1055/s-0043-1767704 37795464 PMC10547534

[13] Ioannidis O Papaemmanouil S Paraskevas G Kotronis A Chatzopoulos S Konstantara A Recurrent small intestine intussusception in a patient with Peutz-Jeghers syndrome. Rev Esp Enferm Dig. (2012) 104(1):37–9. 10.4321/s1130-01082012000100009 22300117

[14] Kirakosyan E Lokhmatov M. High-tech diagnostic methods and enteroscopic treatment of children with Peutz-Jeghers syndrome. Eur J Pediatr Surg. (2020) 30:529–35. 10.1055/s-0039-3400286 31770782

[15] Tong Q. Current research status of Peutz-Jeghers syndrome in children. Zhongguo Dang Dai Er Ke Za Zhi. (2024) 26(10):1122–6. 10.7499/j.issn.1008-8830.2404054 39467684 PMC11527405

[16] Verma A Kanneganti P Kumar B Upadhyaya VD Mandelia A Naik PB Peutz-Jeghers syndrome: management for recurrent intussusceptions. Pediatr Surg Int. (2024) 40(1):148. 10.1007/s00383-024-05723-y 38825635

[17] Dofuku M Yano T Yokoyama K Okada Y Kumagai H Tajima T Management of pediatric Peutz-Jeghers syndrome: highlighting the efficacy and safety of endoscopic ischemic polypectomy. J Pediatr Gastroenterol Nutr. (2025) 80(3):408–16. 10.1002/jpn3.12458 39760314

[18] Wagner A Aretz S Auranen A Bruno MJ Cavestro GM Crosbie EJ The management of Peutz-Jeghers syndrome: European hereditary tumour group (EHTG) guideline. J Clin Med. (2021) 10(3):473. 10.3390/jcm10030473 33513864 PMC7865862

[19] Latchford A Cohen S Auth M Scaillon M Viala J Daniels R Management of Peutz-Jeghers syndrome in children and adolescents: a position paper from the ESPGHAN polyposis working group. J Pediatr Gastroenterol Nutr. (2019) 68(3):442–52. 10.1097/MPG.0000000000002248 30585892

[20] Goneidy A Ross AR Roberts R Hyer W Choudhry M. Laparoscopic-assisted polypectomy: a promising minimally-invasive solution for endoscopically irresectable polyps in children. J Pediatr Surg. (2025) 60(7):162329. 10.1016/j.jpedsurg.2025.162329 40216325

